# Case report: Focal segmental glomerulosclerosis in a pediatric atypical progeroid syndrome

**DOI:** 10.3389/fped.2022.1032653

**Published:** 2022-10-31

**Authors:** Seoyun Jang, Yo Han Ahn, Jung Min Ko, Jae Sung Ko, Sojung Lim, Hee Gyung Kang

**Affiliations:** ^1^ Department of Pediatrics, Seoul National University Children's Hospital, Seoul, South Korea; ^2^ Department of Pediatrics, Seoul National University College of Medicine, Seoul, South Korea; ^3^ Kidney Research Institute, Medical Research Center, Seoul National University College of Medicine, Seoul, South Korea; ^4^ Rare Disease Center, Seoul National University Hospital, Seoul, South Korea; ^5^ Department of Pathology, Seoul National University Hospital, Seoul National University College of Medicine, Seoul, South Korea; ^6^ Wide River Institute of Immunology, Seoul National University, Hongcheon, South Korea

**Keywords:** focal segmental glomerular sclerosis (FSGS), atypical progeroid syndrome, lipodystrophy, LMNA, TGF - β1

## Abstract

Atypical progeroid syndrome (APS) is a rare type of progeroid syndrome mainly caused by heterozygous missense mutations in the *LMNA* (MIM 150330) gene. APS has heterogeneous clinical manifestations, and its kidney manifestations, particularly in children, are rarely documented. Here, we report the first pediatric case of APS with focal segmental glomerulosclerosis (FSGS). A 10-year-old boy with progeroid features was referred to the nephrology clinic because of hyperuricemia. He had dark skin, protruding eyes, and beaked nose and was very thin, suggesting lipodystrophy. He had been treated for recurrent urinary tract infection during infancy, and liver biopsy for persisting hepatitis showed steatohepatitis. He also had hypertrophic cardiomyopathy (HCMP) with mitral and tricuspid valve regurgitation. Genetic studies were performed considering his multisystem symptoms, and he was diagnosed as having APS according to exome sequencing findings (c.898G > C, *p*.Asp300His of *LMNA*). During the first visit to the nephrology clinic, he had minimal proteinuria (urine protein/creatinine ratio of 0.23 mg/mg), which worsened during follow-up. In three years, his urine protein/creatinine ratio and N-acetyl-b-D-glucosaminidase/creatinine ratio increased to 1.52 and 18.7, respectively. The kidney biopsy result was consistent with findings of FSGS, peri-hilar type, showing segmental sclerosis of 1 (5%) glomerulus out of 21 glomeruli. An angiotensin receptor blocker was added to manage his proteinuria. This is the first pediatric report of FSGS in an APS patient with confirmed *LMNA* defect, who manifested progeroid features, lipodystrophy, HCMP with heart valve dysfunction, and steatohepatitis. Our case suggests that screening for proteinuric nephropathy is essential for managing APS patients since childhood.

## Introduction

Progeroid syndromes are a group of rare genetic disorders characterized by clinical features that mimic physiologic aging. Progeroid syndromes share similar clinical features such as hair loss, short stature, skin tightness, cardiovascular diseases, and osteoporosis. However, the underlying mechanism can vary according to the causative gene (1). The progeroid syndrome can be classified into two groups according to its molecular pathophysiology: alterations in components of the nuclear envelope and mutations in genes involved in DNA-repair pathways (1, 2). The nuclear envelope component involved in progeroid syndrome is the nuclear lamina, a thin protein meshwork between chromatin and the inner nuclear membrane (3, 4), composed of lamins. Lamins contribute to the maintenance of nuclear shape and structure, chromatin organization, and other aspects of nuclear metabolism by interacting with regulatory molecules (5–8). Lamin A and lamin C (encoded by *LMNA*, MIM 150330) are widely expressed in somatic cells, and diverse *LMNA* mutations cause various disorders, laminopathies, including diseases affecting striated and cardiac muscle, lipodystrophy syndromes such as familial partial lipodystrophy (FPLD), peripheral neuropathy, and premature aging (progeroid syndromes) (9, 10).

Among the laminopathies, FPLD encompasses abnormal fat distribution and insulin resistance disorders. FPLD type 2 (Dunnigan-type, MIM 151660) is known to be caused by *LMNA* mutation and is characterized by the progressive lipoatrophy of the limbs, buttocks, and trunk sparing the neck and face. Metabolic alterations are common and cardiovascular comorbidities, and hepatic steatosis is often reported (11). Hutchinson-Gilford progeria syndrome (HGPS, MIM 176670) is another form of laminopathy, an early-onset premature aging disorder. It typically presents at 1–2 years of age with severe growth retardation, lipodystrophy, and skeletal and cardiovascular features (12, 13). The average life span is 13 years (12, 14, 15). Typical HGPS is caused by *de novo* heterozygous silent mutation in the *LMNA* gene c.1924C > T (*p*.Gly608Gly) (16, 17). This activates a cryptic splicing site and results in abnormal splicing of the prelamin A, producing a truncated protein called progerin, which is known to cause toxic effects when accumulated (18–21). Atypical progeroid syndrome (APS) is another type of progeria from *LMNA* mutation. APS is very rare, and until now, 69 patients with *LMNA* mutation have been reported worldwide (22–33). It is characterized by not having an accumulation of the Lamin A precursors (14, 15) and is caused by heterozygous *LMNA* mutations other than c.1924C > T. The onset of APS symptoms is relatively late, and the life span of APS patients is generally longer than those of HGPS (12, 14, 15). Though its clinical phenotypes have not been well established, typically, affected patients have growth retardation, joint contractures, and progeroid features, including a prominent nose with beaking, partial alopecia, dental crowding, and skin anomalies (14, 15, 34). Also, most of the patients suffered from marked metabolic abnormalities such as insulin resistance, diabetes mellitus, hypertriglyceridemia, steatohepatitis, various degrees of lipodystrophy, and cardiomyopathy (15, 35, 36).

Kidney involvement as proteinuria has been seldomly described in APS (26), and the pathophysiology of kidney manifestations remains unclear. Here, we report a case of a clinical presentation of an APS patient who presented with proteinuria and pathologically confirmed focal segmental glomerulosclerosis (FSGS).

## Case presentation

A 10-year-old boy visited the nephrology clinic for further evaluation of incidentally found hyperuricemia. Previously, his perinatal medical history was unremarkable, with full-term vaginal delivery and birth weight of 3.64 kg, but he had four episodes of urinary tract infection during infancy. Bilateral vesicoureteral reflux (left grade 4 and right grade 1) was identified at three months, and he was given nitrofurantoin prophylaxis until 22 months. It was discontinued as there was no further urinary tract infection for a year. After the discontinuation, his vesicourethrogram at the age of 28 months showed no reflux. He was the only child, devoid of any remarkable family history of diseases. At 2 years of age, he complained of discoloration of extremities after exposure to cold, but evaluations for autoimmune disease and arteriography of upper and lower extremities did not reveal any abnormal findings. At 7 years of age, he was noticed to have waddling gait and knee and hip flexion limitations. Two years later, marked coxa valga with relative coxa magna with a small pelvis were found on orthopedic evaluation. He visited a genetic specialist at 9 years of age. While he had normal growth with a height of 143 cm (88.6 percentile) and weight of 33 kg (47.5 percentile), with normal development, decreased subcutaneous fat tissue in his trunk and both extremities with pale and dark skin were noted. He also had acanthosis nigricans at his neck and both axillary areas, a short neck, protruding eyes, and a beaked nose. Raynaud's phenomenon was still present. Liver function abnormality was found with elevated liver transaminases (AST 102 IU/L, normal range 15–50IU/L; ALT 248 IU/L, normal range 5–45 IU/L) and cholesterol (LDL-cholesterol 186 mg/dl, normal range 60–140 mg/dl, triglyceride 305 mg/dl, normal range 31–108 mg/dl; HDL-cholesterol 42 mg/dl, normal range: ≥40 mg/dl). His HbA1c was 6.5% (normal range: 4.0–6.4%), with a fasting blood glucose level of 83 mg/dl (normal range: 70–99 mg/dl), and increased insulin (94 μIU/ml, normal range 1.9–15.97 *μ*IU/ml). He was referred to the endocrinologist for elevated HbA1c and dyslipidemia, however, his fasting glucose was lower than 100 mg/dl, but HbA1c and insulin were still elevated (6.1%, 55.7 μIU/ml) showing insulin resistance. Liver biopsy at 10 years showed a fatty change of hepatocytes and portal and periportal fibrosis. Mild mitral and tricuspid valve regurgitation was noted, which worsened during follow-up along with the development of hypertrophic cardiomyopathy (HCMP), requiring mechanical mitral valve replacement at 13 years of age. His diagnosis remained elusive despite multisystem symptoms. Various studies targeting progressive storage disorders, including glycogen storage disease, were performed, with no meaningful results. Eventually, he was found to have a *de novo* heterozygous mutation c.898G > C (*p*.Asp300His) of *LMNA*, genetically diagnosing the patient as APS. During regular follow-up for the steatohepatitis, hyperuricemia (serum uric acid 7.6 mg/dl, normal range: 3.0–7.0 mg/dl) was noticed, and he was referred to the nephrology clinic.

**Figure 1:**
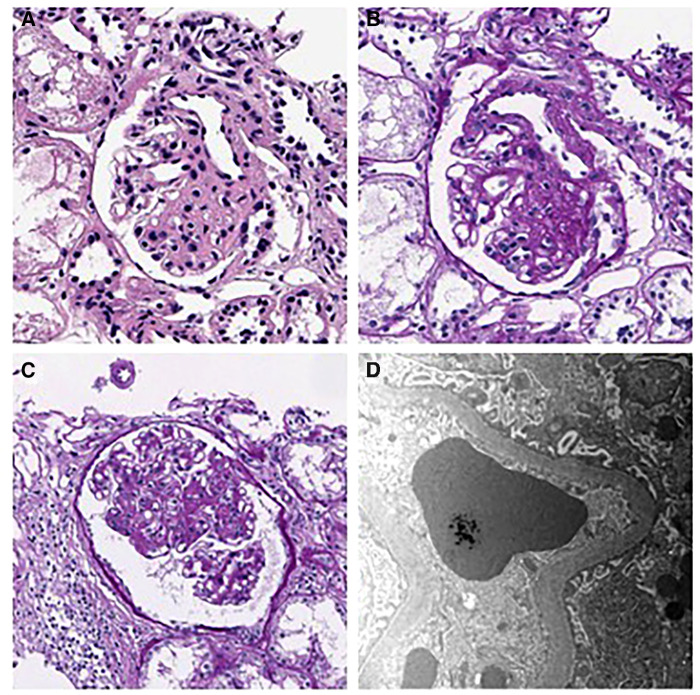
Kidney biopsy findings. (**A–B**) H&E and PAS staining show segmental sclerosis at the vascular pole of the glomerulus. The patient was diagnosed as having focal segmental glomerulosclerosis, perihilar variant. (**A**) H&E stain, × 400, (**B**) PAS stain, × 400). (**C**) Mild endocapillary hypercellularity and glomerular enlargement was observed in other glomeruli, with a maximum diameter of 302 μm. (PAS stain, × 250). (**D**) Electron microscopy showing mild effacement of foot processes. (EM, × 12,000).

His hyperuricemia was managed well with benzbromarone. Initially, minimal proteinuria [urine protein/creatinine (Cr) ratio of 0.23 mg/mg] was present, which gradually worsened during follow-up. At 13, his urine protein/Cr ratio (normal range: 0–0.2 mg/mg) and N-acetyl-b-D-glucosaminidase/Cr ratio (normal range: 0–5.6 IU/gCr) increased to 1.52 and 18.7, respectively. Urine ß2 microglobulin was 0.33 ug/ml, and serum uric acid level was 12.4 mg/dl. However, he was normotensive with a height of 160.4 cm (50–75 percentile), a weight of 37.7 kg (5–10 percentile), and a BMI of 14.65 kg/m^2^, with normal serum albumin (4.4 g/dl, normal range: 3.3–5.2 g/dl) and serum Cr (0.71 mg/dl, normal range: 0.31–0.88 mg/dl). Doppler kidney sonography was unremarkable with normal size, parenchymal echogenicity, and intact internal perfusion. His kidney biopsy revealed FSGS, peri-hilar type, showing segmental sclerosis of 1 (5%) glomerulus out of 21 glomeruli. In microscopic findings, glomerular size was mildly increased, and there was focal mild hypercellularity involving mesangial and endothelial cells (Figure 1). Tubules were slightly atrophic, and there was no interstitial fibrosis, with mild focal infiltration of mononuclear cells. Immunofluorescence staining for immunoglobulins (IgG, IgM, and IgA), Kappa light chains, Lambda light chains, complement C3 and C1q, were all negative. Electron microscopy revealed a normal glomerular basement membrane, and effacement of the foot process was mild. There were no electron-dense deposits.

**Figure 2:**
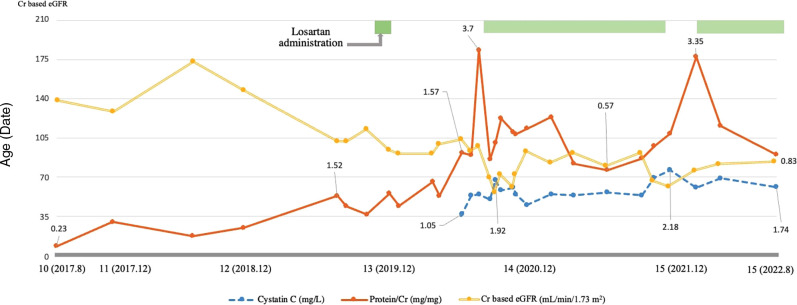
Characteristics of the patient during the follow-up. This graph shows the patient's trend of proteinuria and creatinine-based estimated glomerular filtration rate (eGFR) and cystatin C levels during the follow-up. Proteinuria is indicated using a solid line, cystatin C using a dashed line, and creatinine-based eGFR using a double line. The patient's proteinuria and kidney function waxed and waned during the follow-up, and following the administration of losartan, his proteinuria and renal function improved and remains stationary. Creatinine based eGFR (ml/min/1.73 m^2^), Cystatin C (mg/L), Protein/Cr (mg/mg).

An Angiotensin receptor blocker (Losartan) was added to manage his proteinuria (0.7 mg/kg), but it was difficult to continue due to dizziness. Since his proteinuria worsened during follow-up (urine protein/Cr ratio: 3.7 mg/mg, serum Cr 0.69 mg/dl, eGFR 98.22 ml/min/1.73 m^2^), losartan was restarted. His proteinuria waxed and waned with his cardiac condition, and the dosage was adjusted according to the symptoms (up to 1.2 mg/kg) (Figure 2). While taking losartan, his proteinuria did not aggravate, and his kidney function stayed stationary (urine protein/Cr ratio: 1.34 mg/mg, serum Cr 0.83 mg/dl, eGFR 82.45 ml/min/1.73 m^2^). During his last follow-up at 15 years of age, he did well without complaints and worked out daily. He has been prescribed benzbromarone, losartan, and sodium bicarbonate at the nephrology clinic (serum uric acid 6.8 mg/dl, serum Cr 0.79 mg/dl, cystatin C 1.74 mg/l, eGFR 84.43 ml/min/1.73 m^2^, and urine protein/Cr ratio 0.83 mg/mg). He has taken warfarin for mitral valve replacement and a pacemaker for postoperative sinus node dysfunction. He was also taking a beta-blocker, diuretic, and amlodipine for HCMP and omega-3 for hyperlipidemia.

## Discussion

**Figure 3:**
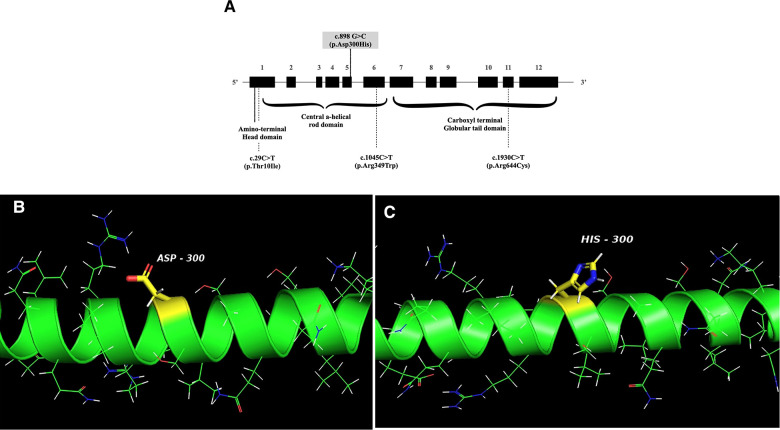
(**A**) Schematic structure of the LMNA gene. The mutation of the patient is indicated with a solid line, and the locations of mutations associated with FSGS are shown in dotted lines. FSGS: focal segmental glomerular sclerosis. (**B**,**C**) The crystal structure of wild-type human lamin A coil 2B domain and a model of proposed *p*.Asp300His mutant. I-TASSER software and PyMol were used to model the wild-type human lamin A and the *p*.Asp300His mutant. The peptide chain of the human lamin A coil 2B domain (aa. 290–310 from left to right) is shown as an alpha-helical structure (green). Side chains are shown as sticks. The carbon atoms in the mutation site are highlighted in yellow and other carbons are in green. Nitrogen atoms are labeled blue, oxygen atoms are red, hydrogen atoms are white, and sulfur atoms are shown orange.

This is the first report of FSGS in a pediatric APS patient with confirmed *LMNA* defect, who manifested progeroid features, lipodystrophy, HCMP with heart valve dysfunction, and steatohepatitis. APS is an extremely rare disease, and kidney involvement in APS is not a typical finding. Previous reports of kidney manifestation showed proteinuria, including the nephrotic range (26). While the mutation of our case, c.898G > C (*p*.Asp300His) in *LMNA* (Figure 3), was previously reported in two cases (25, 29), kidney involvement was not described. It is not a novel mutation, as the mutation was reported previously (25, 29). However, it is an extremely rare mutation, so its frequency has not been reported in genomic databases, including GNOMAD or Clinvar. One case with this variant was a 24-year-old Chinese man with multiple vascular lesions, progeroid features, hypertension, numerous intracranial calcifications, peripheral artery disease, and dyslipidemia (29). The other case is a 23-year-old woman from Myanmar with progeroid features, including short stature, thin scalp hair, absent eyebrow and eyelashes, and a beaked nose. She suffered from hypertension, secondary amenorrhea, generalized lipodystrophy, bilateral carotid artery stenosis, and left ventricular hypertrophy (25). Another missense variant of the same nucleotide locus (c.898G > A, *p*.Asp300Asn) was found in a 31-year-old French man with progeroid features, osteoporosis, premature atheromatosis, lipoatrophy, and cerebral ischemic disease (28). APS with cardiovascular diseases and dyslipidemia were common in all these patients, including our patient, but proteinuria was not described. Therefore, it is unclear if the FSGS of our case is a true manifestation of his APS.

However, proteinuria has been reported in several cases with *LMNA* mutations, and to our knowledge, nine patients were confirmed with FSGS by kidney biopsy (23, 24, 26, 37–41) (Table 1). The primary diagnosis of these cases was lipodystrophy with or without APS. Their presentations of proteinuria were all during adulthood, and two of them with APS features required kidney replacement therapy (23, 26). Since the others with the same mutation (*p*.Arg349Trp) had normal to impaired kidney function, the kidney outcome seems variable (23, 26, 37, 41), and proteinuria might have been present since their childhood as in our case, but not detected earlier. Therefore, if possible, our patient also needs careful follow-up and intervention since some required dialysis in their 30s, although their variant site was different from ours.

**Table 1:** *LMNA* mutations associated with pathologic findings of focal segmental glomerulosclerosis.

N	Literature, year of publication	*LMNA* mutation	Sex	Age at diagnosis of kidney disease (years)	Clinical features			Kidney parameters at the time of biopsy	Remarks
					Progeroid features	Lipodystrophy	Others		
1	Rankin et al. (40), 2008	c.1930C > T (*p*.Arg644Cys)	F	32	N/D	FPLD, Dunnigan variety	DM, HTN, dyslipidemia	N/D	
2	Thong et al. (41), 2013	c.1045C > T (*p*.Arg349Trp)	F	35	N/D	FPLD, Non-Dunnigan	HTN, AR, paroxysmal atrial fibrillation	UPR 595 mg/mmol, sALB 27 g/L, eGFR 33 ml/min/1.73 m^2^, sCr 1.7 mg/dl	
3	Thong et al. (41), 2013	c.1045C > T (*p*.Arg349Trp)	F	27	N/D	FPLD, Non-Dunnigan	HTN, mild MR, trivial AR, bilateral hearing loss	UPR 3.67 g/24 h, sALB 35 g/LL, eGFR 141 ml/min/1.73 m^2^, sCr 0.52 mg/dl	
4	Thong et al. (41), 2013	c.1045C > T (*p*.Arg349Trp)	M	35	N/D	FPLD, Non-Dunnigan	Cardiomyopathy, dyslipidemia, erectile dysfunction, Rt. Hearing loss	UPR 123 mg/mmol, sALB 38 g/L, eGFR 115 ml/min/1.73 m^2^, sCr 0.77 mg/dl	
5	Thong et al. (41), 2013	c.1045C > T (*p*.Arg349Trp)	F	40	N/D	FPLD, Non-Dunnigan	DM, HTN, cardiomyopathy, 1st degree heart block, bilateral hearing loss	UPR 121 mg/mmol, sALB 41 g/L, eGFR 73 ml/min/1.73 m^2^, sCr 0.7 mg/dl	
6	Hussain et al. (24), 2018	c.29C > T (*p*.Thr10Ile)	F	33	N/D	Generalized lipodystrophy	DM, dyslipidemia, AR, MR, TR, heart failure, left ventricular hypertrophy, atherosclerosis, primary amenorrhea, steatohepatitis,	UPR >2 g/day	
7	Fountas et al. (37), 2017	c.1045C > T (*p*.Arg349Trp)	F	27	N/D	FPLD, Non-Dunnigan	DM, dyslipidemia, myopathy, hepatic steatosis	UPR 2.2 g/24 h, sALB 4.2 g/dl, eGFR 118 ml/min/1.73 m^2^, sCr 0.67 mg/dl	USG normal, ACEi improved kidney function, proteinuria (sCr 0.68 mg/dl, 117 ml/min/1.73 m^2^, UPR 1.1 g/24hr)
8	Magno et al. (26), 2020	c.1045C > T (*p*.Arg349Trp)	M	34	beaked nose, prominent eyes, partial alopecia, skin atrophy, thin lips, small mandible	FPLD, Non-Dunnigan	HTN, dyslipidemia, moderate MR, TR, neuroendocrine tumor, sensorineural hearing loss, hepatic steatosis,	UPR 9 g/24 h, eGFR 30 ml/min/1.73 m^2^, sCr 7 mg/dl	HD, KT
9	Hussain et al. (23), 2020	c.1045C > T (*p*.Arg349Trp)	F	38	Pointed nose, thin lips	FPLD, Non-Dunnigan	DM, HTN, dyslipidemia, cardiomyopathy, mild AR, MR, TR	UPR 4.5 g/24 h	dialysis

N/D, not described; DM, diabetes mellitus; HTN, hypertension; AR, aortic regurgitation; MR, mitral regurgitation; TR, tricuspid regurgitation; UPR, urine protein; sALB, serum albumin; eGFR, estimated GFR; sCr, serum creatinine; HD, hemodialysis; KT, kidney transplantation; USG, ultrasonography.

However, the pathophysiology of FSGS in *LMNA* mutations remains unclear. Transforming growth factor-beta1 (TGFβ1), which is activated in lipodystrophy in laminopathies (42, 43), might play a role in the disease mechanism. It is well known that activation of TGFβ1, the central regulator of fibrotic responses (44, 45), leads to mesangial cell matrix overproduction and glomerulosclerosis (46, 47) in diseased glomeruli. Interestingly, lamin A or C was essential for inhibiting fibroblast proliferation by TGFβ1 (48). Therefore, *LMNA* mutation might be linked to FSGS. Also, metabolic alteration of laminopathies, including our patient, might contribute to podocyte injury leading to kidney damage (49, 50, 51). Our patient also had a history of urinary tract infection and vesicoureteral reflux; FSGS might come from reflux nephropathy. However, his kidney pathology was incompatible with typical pathologic findings of reflux nephropathy, such as interstitial scarring, tubular atrophy, or loss of nephron mass.

Regarding the treatment, as TGFβ1 plays a crucial role in pathogenesis, targeting this cytokine appears promising. However, its therapeutic application is held back because of its multifunctional and pleiotropic actions. Fresolimumab, a human monoclonal antibody neutralizing human isoforms of TGFβ, was proven ineffective in clinical trials in FSGS (52, 53). Other approaches, including decreasing the production of prelamin A or clearing progerin (54) showed limited effect (55), as APS is not associated with the accumulation of lamin A precursors (15, 56). Therefore, so far, early recognition and treatment of the manifestations is the mainstay of treatment, which makes identifying the phenotypes of the disease more crucial. Therefore, in this case, early recognition and intervention might improve kidney outcomes.

In conclusion, this is the first pediatric APS patient with FSGS. Though kidney manifestation of the disease has not been emphasized before, accompanying proteinuria and FSGS might further deteriorate the prognosis, especially when detected belatedly after the advancement of sclerosis. Therefore, screening for proteinuria and kidney function should be considered when managing patients with APS. Further studies are needed for novel treatment strategies.

## Data Availability

The original contributions presented in the study are included in the article/Supplementary Material, further inquiries can be directed to the corresponding author/s.

## References

[1] Carrero D Soria-Valles C López-Otín C. Hallmarks of progeroid syndromes: lessons from mice and reprogrammed cells. Dis Model Mech. (2016) 9:719–35. 10.1242/dmm.024711 27482812 PMC4958309

[2] Navarro CL Cau P Lévy N. Molecular bases of progeroid syndromes. Hum Mol Genet. (2006) 15(Spec No 2):R151–61. 10.1093/hmg/ddl214 16987878

[3] Dwyer N Blobel G. A modified procedure for the isolation of a pore complex-lamina fraction from rat liver nuclei. J Cell Biol. (1976) 70:581–91. 10.1083/jcb.70.3.581 986398 PMC2109848

[4] Gerace L Huber MD. Nuclear lamina at the crossroads of the cytoplasm and nucleus. J Struct Biol. (2012) 177:24–31. 10.1016/j.jsb.2011.11.007 22126840 PMC3261324

[5] Burke B Stewart CL. The nuclear lamins: flexibility in function. Nat Rev Mol Cell Biol. (2013) 14:13–24. 10.1038/nrm3488 23212477

[6] Han X Feng X Rattner JB Smith H Bose P Suzuki K Tethering by lamin A stabilizes and targets the ING1 tumour suppressor. Nat Cell Biol. (2008) 10:1333–40. 10.1038/ncb1792 18836436

[7] Ivorra C Kubicek M González JM Sanz-González SM Alvarez-Barrientos A O’Connor JE A mechanism of AP-1 suppression through interaction of c-Fos with lamin A/C. Genes Dev. (2006) 20:307–20. 10.1101/gad.349506 16452503 PMC1361702

[8] Wilson KL Foisner R. Lamin-binding proteins. Cold Spring Harb Perspect Biol. (2010) 2:a000554. 10.1101/cshperspect.a000554 20452940 PMC2845209

[9] Worman HJ Ostlund C Wang Y. Diseases of the nuclear envelope. Cold Spring Harb Perspect Biol. (2010) 2:a000760. 10.1101/cshperspect.a000760 20182615 PMC2828284

[10] Worman HJ Bonne G. “Laminopathies”: a wide spectrum of human diseases. Exp Cell Res. (2007) 313:2121–33. 10.1016/j.yexcr.2007.03.028 17467691 PMC2964355

[11] Fernandez-Pombo A Sanchez-Iglesias S Araujo-Vilar D Guillin-amarelle C. Lipodystrophic laminopathies diagn clues. Nucleus. (2018) 9:249–60. 10.1080/19491034.2018.1454167 29557732 PMC5973260

[12] Hennekam RC. Hutchinson-Gilford progeria syndrome: review of the phenotype. Am J Med Genet A. (2006) 140:2603–24. 10.1002/ajmg.a.31346 16838330

[13] Merideth MA Gordon LB Clauss S Sachdev V Smith AC Perry MB Phenotype and course of Hutchinson-Gilford progeria syndrome. N Engl J Med. (2008) 358:592–604. 10.1056/NEJMoa0706898 18256394 PMC2940940

[14] Doubaj Y De Sandre-Giovannoli A Vera EV Navarro CL Elalaoui SC Tajir M An inherited LMNA gene mutation in atypical progeria syndrome. Am J Med Genet A. (2012) 158A:2881–7. 10.1002/ajmg.a.35557 22991222

[15] Garg A Subramanyam L Agarwal AK Simha V Levine B D’Apice MR Atypical progeroid syndrome due to heterozygous missense LMNA mutations. J Clin Endocrinol Metab. (2009) 94:4971–83. 10.1210/jc.2009-0472 19875478 PMC2795646

[16] De Sandre-Giovannoli A Bernard R Cau P Navarro C Amiel J Boccaccio I Lamin a truncation in Hutchinson-Gilford progeria. Science. (2003) 300:2055. 10.1126/science.1084125 12702809

[17] Eriksson M Brown WT Gordon LB Glynn MW Singer J Scott L Recurrent de novo point mutations in lamin A cause Hutchinson-Gilford progeria syndrome. Nature. (2003) 423:293–8. 10.1038/nature01629 12714972 PMC10540076

[18] Dechat T Shimi T Adam SA Rusinol AE Andres DA Spielmann HP Alterations in mitosis and cell cycle progression caused by a mutant lamin A known to accelerate human aging. Proc Natl Acad Sci. (2007) 104:4955–60. 10.1073/pnas.0700854104 17360326 PMC1829246

[19] Delbarre E Tramier M Coppey-Moisan M Gaillard C Courvalin JC Buendia B. The truncated prelamin A in Hutchinson-Gilford progeria syndrome alters segregation of A-type and B-type lamin homopolymers. Hum Mol Genet. (2006) 15:1113–22. 10.1093/hmg/ddl026 16481358

[20] Liu Y Rusinol A Sinensky M Wang Y Zou Y. DNA Damage responses in progeroid syndromes arise from defective maturation of prelamin A. J Cell Sci. (2006) 119:4644–9. 10.1242/jcs.03263 17062639 PMC3105909

[21] Shumaker DK Dechat T Kohlmaier A Adam SA Bozovsky MR Erdos MR Mutant nuclear lamin A leads to progressive alterations of epigenetic control in premature aging. Proc Natl Acad Sci. (2006) 103:8703–8. 10.1073/pnas.0602569103 16738054 PMC1472659

[22] Ajluni N Meral R Neidert AH Brady GF Buras E McKenna B Spectrum of disease associated with partial lipodystrophy: lessons from a trial cohort. Clin Endocrinol (Oxf). (2017) 86:698–707. 10.1111/cen.13311 28199729 PMC5395301

[23] Hussain I Jin RR Baum HBA Greenfield JR Devery S Xing C Multisystem progeroid syndrome with lipodystrophy, cardiomyopathy, and nephropathy due to an LMNA p.R349W variant. J Endocr Soc. (2020) 4:bvaa104. 10.1210/jendso/bvaa104 32939435 PMC7485795

[24] Hussain I Patni N Ueda M Sorkina E Valerio CM Cochran E A novel generalized lipodystrophy-associated progeroid syndrome due to recurrent heterozygous LMNA p.T10I mutation. J Clin Endocrinol Metab. (2018) 103:1005–14. 10.1210/jc.2017-02078 29267953 PMC6283411

[25] Kandhaya-Pillai R Hisama FM Bucks SA Yarzar S Korovou H Martin GM Novel LMNA mutations in Greek and Myanmar patients with progeroid features and cardiac manifestations. Aging Pathobiol Ther. (2020) 2:101–5. 10.31491/apt.2020.06.021 32954377 PMC7500617

[26] Magno S Ceccarini G Pelosini C Ferrari F Prodam F Gilio D Atypical progeroid syndrome and partial lipodystrophy due to LMNA gene p.R349W mutation. J Endocr Soc. (2020) 4:bvaa108. 10.1210/jendso/bvaa108 32913962 PMC7474543

[27] Motegi S Yokoyama Y Uchiyama A Ogino S Takeuchi Y Yamada K First Japanese case of atypical progeroid syndrome/atypical Werner syndrome with heterozygous LMNA mutation. J Dermatol. (2014) 41:1047–52. 10.1111/1346-8138.12657 25327215

[28] Renard D Fourcade G Milhaud D Bessis D Esteves-Vieira V Boyer A Novel LMNA mutation in atypical werner syndrome presenting with ischemic disease. Stroke. (2009) 40:e11–4. 10.1161/STROKEAHA.108.531780 19095983

[29] Yanhua X Suxian Z. Cerebral haemorrhage in a young patient with atypical Werner syndrome due to mutations in LMNA. Front Endocrinol (Lausanne). (2018) 9:433. 10.3389/fendo.2018.00433 30123186 PMC6085819

[30] Jiajue R Feng K Wang R Xia W. Recurrent femoral fractures in a boy with an atypical progeroid syndrome: a case report. Calcif Tissue Int. (2020) 106:325–30. 10.1007/s00223-019-00639-5 31807803

[31] Schultz B Miller DD Maguiness S. Diffuse, mottled hyperpigmentation and mutations in LMNA gene in a 5-year-old boy, his mother, and his grandmother: atypical progeroid syndrome. Pediatr Dermatol. (2019) 36:913–7. 10.1111/pde.13917 31378009

[32] Yukina M Nuralieva N Sorkina E Troshina E Tiulpakov A Belaya Z Atypical progeroid syndrome (p.E262K LMNA mutation): a rare cause of short stature and osteoporosis. Endocrinol Diabetes Metab Case Rep. (2021) 2021:20-0188. 10.1530/EDM-20-0188 PMC8052577 33859056

[33] Lee S Park SM Kim HJ Kim JW Yu DS Lee YB. Genomic diagnosis by whole genome sequencing in a Korean family with atypical progeroid syndrome. J Dermatol. (2015) 42:1149–52. 10.1111/1346-8138.13005 26122271

[34] Liang L Zhang H Gu X. Homozygous LMNA mutation R527C in atypical Hutchinson-Gilford progeria syndrome: evidence for autosomal recessive inheritance. Acta Paediatr. (2009) 98:1365–8. 10.1111/j.1651-2227.2009.01324.x 19432833

[35] Doh YJ Kim HK Jung ED Choi SH Kim JG Kim BW Novel LMNA gene mutation in a patient with atypical Werner's Syndrome. Korean J Intern Med. (2009) 24:68–72. 10.3904/kjim.2009.24.1.68 19270485 PMC2687649

[36] Mahdi L Kahn A Dhamija R Vargas HE. Hepatic steatosis resulting from LMNA-associated familial lipodystrophy. ACG Case Rep J. (2020) 7:e00375. 10.14309/crj.0000000000000375 32548202 PMC7224721

[37] Fountas A Giotaki Z Dounousi E Liapis G Bargiota A Tsatsoulis A Familial partial lipodystrophy and proteinuric renal disease due to a missense c.1045C > T LMNA mutation. Endocrinol Diabetes Metab Case Rep. (2017) 2017:17-0049. 10.1530/EDM-17-0049 28620495 PMC5467650

[38] Imachi H Murao K Ohtsuka S Fujiwara M Muraoka T Hosokawa H A case of Dunnigan-type familial partial lipodystrophy (FPLD) due to lamin A/C (LMNA) mutations complicated by end-stage renal disease. Endocrine. (2009) 35:18–21. 10.1007/s12020-008-9127-1 19011997

[39] Owen KR Donohoe M Ellard S Clarke TJ Nicholls AJ Hattersley AT Mesangiocapillary glomerulonephritis type 2 associated with familial partial lipodystrophy (Dunnigan-Kobberling syndrome). Nephron Clin Pract. (2004) 96:c35–8. 10.1159/000076396 14988595

[40] Rankin J Auer-Grumbach M Bagg W Colclough K Nguyen TD Fenton-May J Extreme phenotypic diversity and nonpenetrance in families with the LMNA gene mutation R644C. Am J Med Genet A. (2008) 146A:1530–42. 10.1002/ajmg.a.32331 18478590

[41] Thong KM Xu Y Cook J Takou A Wagner B Kawar B Cosegregation of focal segmental glomerulosclerosis in a family with familial partial lipodystrophy due to a mutation in LMNA. Nephron Clin Pract. (2013) 124:31–7. 10.1159/000354716 24080738

[42] Clouthier DE Comerford SA Hammer RE. Hepatic fibrosis, glomerulosclerosis, and a lipodystrophy-like syndrome in PEPCK-TGF-beta1 transgenic mice. J Clin Invest. (1997) 100:2697–713. 10.1172/JCI119815 9389733 PMC508473

[43] Le Dour C Wu W Béréziat V Capeau J Vigouroux C Worman HJ. Extracellular matrix remodeling and transforming growth factor-beta signaling abnormalities induced by lamin A/C variants that cause lipodystrophy. J Lipid Res. (2017) 58:151–63. 10.1194/jlr.M071381 27845687 PMC5234718

[44] Frangogiannis N. Transforming growth factor-beta in tissue fibrosis. J Exp Med. (2020) 217:e20190103. 10.1084/jem.20190103 32997468 PMC7062524

[45] Baek HS. Mechanism, clinical consequences, and management of dyslipidemia in children with nephrotic syndrome. Child Kidney Dis. (2022) 26(1):25–30. 10.3339/ckd.22.020

[46] Kim JH Kim BK Moon KC Hong HK Lee HS. Activation of the TGF-beta/smad signaling pathway in focal segmental glomerulosclerosis. Kidney Int. (2003) 64:1715–21. 10.1046/j.1523-1755.2003.00288.x 14531804

[47] Lee HS Song CY. Effects of TGF-beta on podocyte growth and disease progression in proliferative podocytopathies. Kidney Blood Press Res. (2010) 33:24–9. 10.1159/000285844 20185928

[48] Van Berlo JH Voncken JW Kubben N Broers JL Duisters R van Leeuwen RE A-type lamins are essential for TGF-beta1 induced PP2A to dephosphorylate transcription factors. Hum Mol Genet. (2005) 14:2839–49. 10.1093/hmg/ddi316 16115815

[49] Chen RX Zhang L Ye W Wen YB Si N Li H The renal manifestations of type 4 familial partial lipodystrophy: a case report and review of literature. BMC Nephrol. (2018) 19:111. 10.1186/s12882-018-0913-6 29747582 PMC5946515

[50] Zhang LH Zhu XY Eirin A Nargesi AA Woollard JR Santelli A Early podocyte injury and elevated levels of urinary podocyte-derived extracellular vesicles in swine with metabolic syndrome: role of podocyte mitochondria. Am J Physiol Ren Physiol. (2019) 317:F12–22. 10.1152/ajprenal.00399.2018 PMC6692726 31042059

[51] Lan HY. Smads as therapeutic targets for chronic kidney disease. Kidney Res Clin Pract. (2012) 31(1):4–11. 10.1016/j.krcp.2011.12.001 26889404 PMC4715089

[52] Trachtman H Fervenza FC Gipson DS Heering P Jayne DR Peters H A phase 1, single-dose study of fresolimumab, an anti-TGF-beta antibody, in treatment-resistant primary focal segmental glomerulosclerosis. Kidney Int. (2011) 79:1236–43. 10.1038/ki.2011.33 21368745 PMC3257033

[53] Vincenti F Fervenza FC Campbell KN Diaz M Gesualdo L Nelson P A phase 2, double-blind, placebo-controlled, randomized study of fresolimumab in patients with steroid-resistant primary focal segmental glomerulosclerosis. Kidney Int Rep. (2017) 2:800–10. 10.1016/j.ekir.2017.03.011 29270487 PMC5733825

[54] Harhouri K Frankel D Bartoli C Roll P De Sandre-Giovannoli A Lévy N. An overview of treatment strategies for Hutchinson-Gilford progeria syndrome. Nucleus. (2018) 9:246–57. 10.1080/19491034.2018.1460045 29619863 PMC5973194

[55] Varela I Pereira S Ugalde AP Navarro CL Suárez MF Cau P Combined treatment with statins and aminobisphosphonates extends longevity in a mouse model of human premature aging. Nat Med. (2008) 14:767–72. 10.1038/nm1786 18587406

[56] Toth JI Yang SH Qiao X Beigneux AP Gelb MH Moulson CL Blocking protein farnesyltransferase improves nuclear shape in fibroblasts from humans with progeroid syndromes. Proc Natl Acad Sci. (2005) 102:12873–8. 10.1073/pnas.0505767102 16129834 PMC1193538

